# Supplementary material for: Dentofacial mini- and microesthetics as perceived by dental students: A cross-sectional multi-site study
Source: PLoS One. 2020 Mar 12;15(3):e0230182. doi: 10.1371/journal.pone.0230182 (PMC7067484; doi:10.1371/journal.pone.0230182)
Supplement: S1 Data — (PDF) [file pone.0230182.s001.pdf]

Dentofacial Aesthetics Instrument for Dental Students

[Please note that this is a demonstration of the structure of the questionnaire. The layout of the version administered to respondents is different.]

**1. Demographic data (3 items)**	
**1.1 Sex**	FEMALE / MALE
**1.2 Age (years)**	
**1.3 Academic year**	1 2 3 4 5
**2. Self-Aesthetics I (multiple choice, 11 items)**	
**2.1 Have you ever avoided smiling because of the appearance of your teeth?**	yes / no
**2.2 How do you smile for photos?**	with a full smile (lips open) / with a partial smile (lips partially open) / with closed lips
**2.3 Have you ever noticed that you were looking at others because you wished you had their smile?**	yes / no
**2.4 Would you like to change your smile?**	yes / no
**2.5 Are you satisfied with the color of your teeth?**	yes / no / partly
**2.6 In your opinion, are the shape and size of your teeth in harmony?**	yes /no / partly
**2.7 Are your teeth crowded?**	yes, in both arches / yes, in one of the arches / no
**2.8 Does the line between your upper central incisors fall in the midline of your face?**	yes / no / I have never observed
**2.9 Does the line between your lower central incisors fall in the midline of your face?**	yes / no / I have never observed
**2.10 Do the lines between your upper and lower incisors fall in the same line?**	yes / no / I have never observed
**2.11 Is your gum visible when you smile?**	yes / no / I have never observed
**3. Self-Aesthetics II (rating, 6 items)**	
**Instruction:** Please indicate your satisfaction with different aspects of your dental and facial aesthetics on a 1 to 5 scale, where 1 means "not at all" and 5 means "absolutely".	
**3.1 How satisfied are you with the shape of your**	1 (not at all) 2 3 4 5 (absolutely)

**teeth?**	
**3.2 How satisfied are you with the size of your teeth?**	1 (not at all) 2 3 4 5 (absolutely)
**3.3 How satisfied are you with the orderliness of your teeth?**	1 (not at all) 2 3 4 5 (absolutely)
**3.4 How satisfied are you with the whiteness of your teeth?**	1 (not at all) 2 3 4 5 (absolutely)
**3.5 How satisfied are you with the harmony between your teeth and lips?**	1 (not at all) 2 3 4 5 (absolutely)
**3.6 How satisfied are you with the aesthetics of your smile in general?**	1 (not at all) 2 3 4 5 (absolutely)
**4. Photo Rating (smile, A to E, 10 items, 5 images per item, with photo album)**	
**4.1-4.10 Choose the image that you find the most attractive.**	A B C D E [with five different images]
