# Supplementary material for: Dentofacial mini- and microesthetics as perceived by dental students: A cross-sectional multi-site study
Source: PLoS One. 2020 Mar 12;15(3):e0230182. doi: 10.1371/journal.pone.0230182 (PMC7067484; doi:10.1371/journal.pone.0230182)

Please note that the images were not shown in this order and layout, this is for illustration only.

Smile 1.: The smile arc changes

A: Tilted 10 degrees upward (slightly inverted)

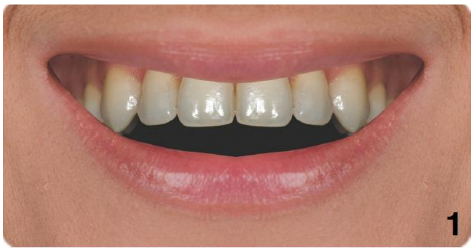

B: Tilted 20 degrees upward (markedly inverted)

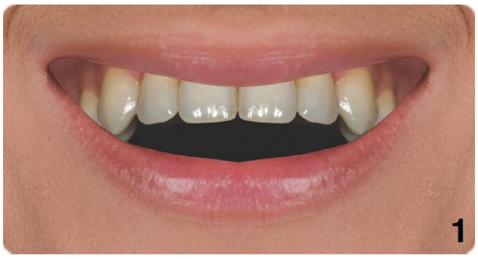

C: Unmodified (plane)

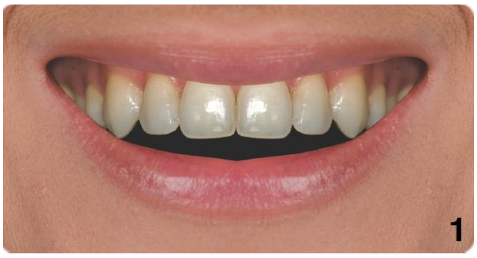

D: Tilted 10 degrees downward (slightly convex)

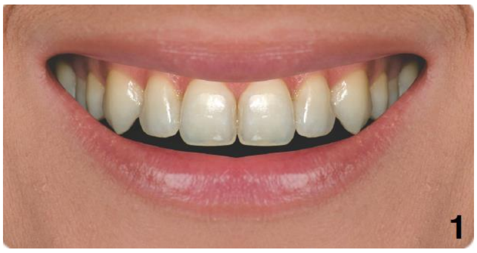

E: Tilted 20 degrees downward (markedly convex)

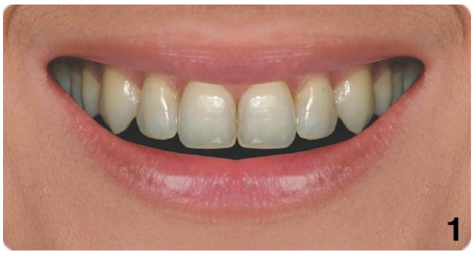

Smile 2. The visibility of the upper gum changes (gingival smile)

A: Both arches shifted 2 mm upward

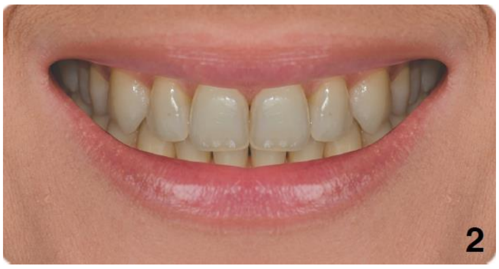

B: Both arches shifted 1 mm upward

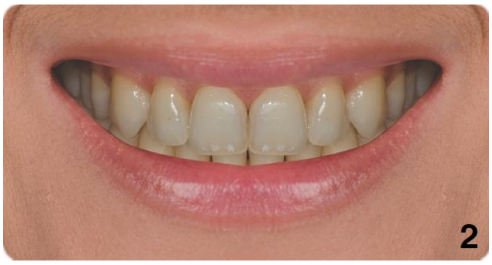

C: Unmodified

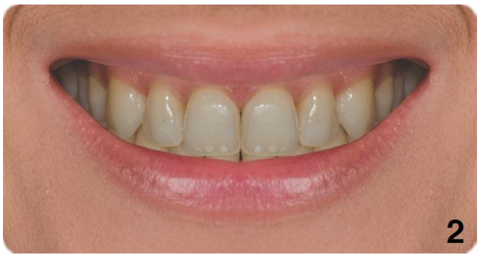

D: Both arches shifted 1 mm downward

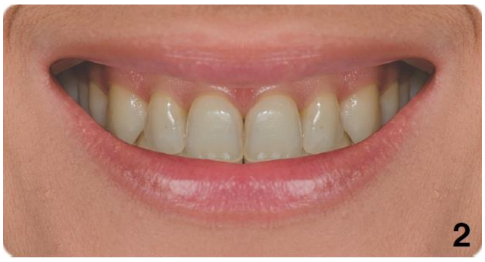

E: Both arches shifted 2 mm downward

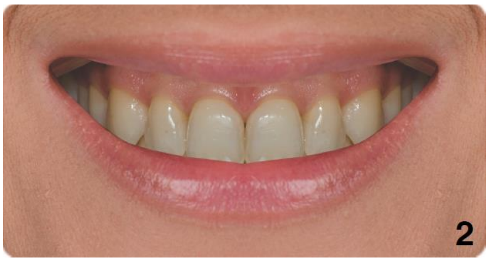

Smile 3. The length of canines and lateral incisors changes

A: unmodified

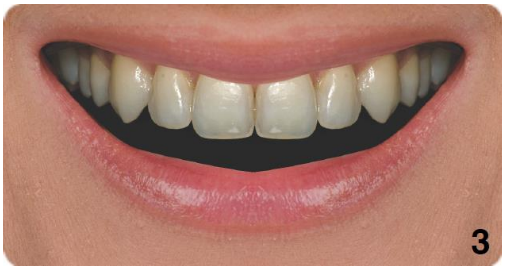

B: canines shorter by 1 mm

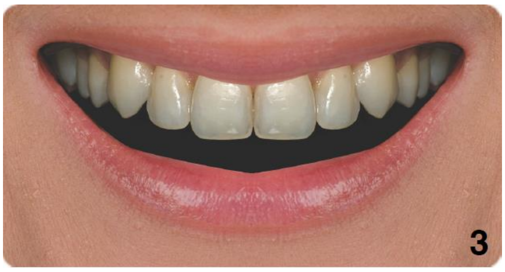

C: canines shorter by 1 mm, lateral incisors longer by 1 mm

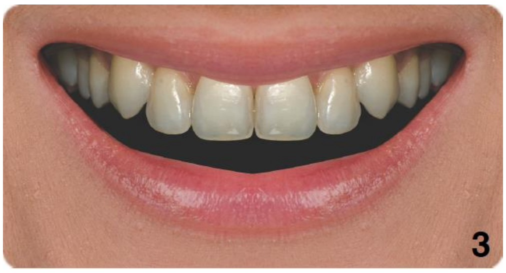

D: canines longer by 1 mm

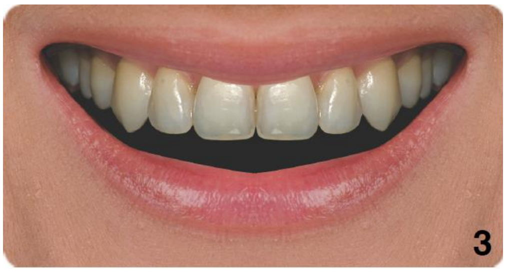

E: both lateral incisors and canines longer by 1 mm

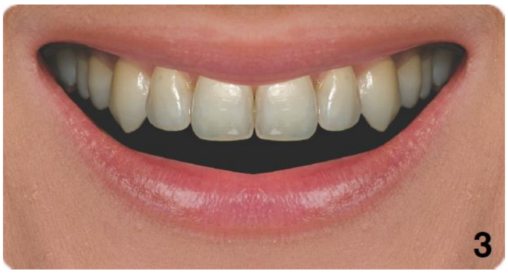

Smile 4. The width of the buccal corridor changes

A: unmodified

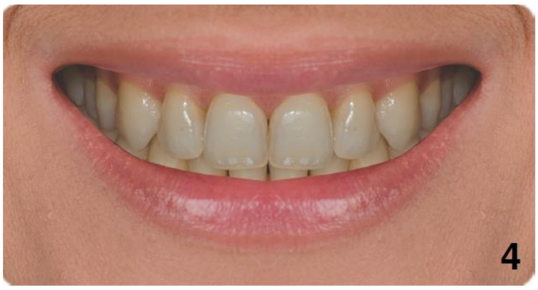

B: +1 mm

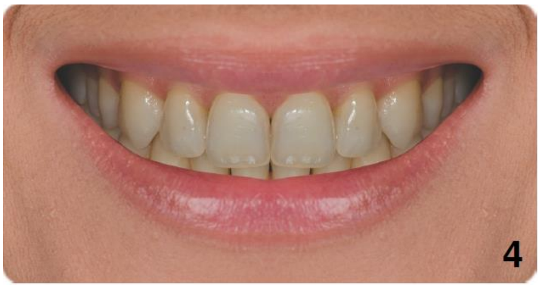

C: +2 mm

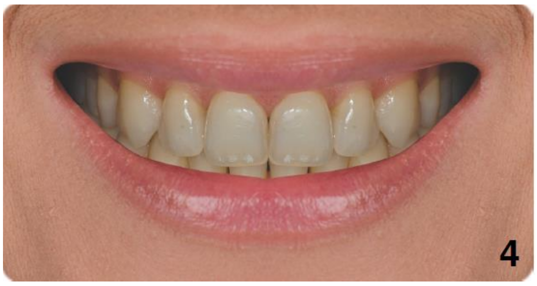

D: +3 mm

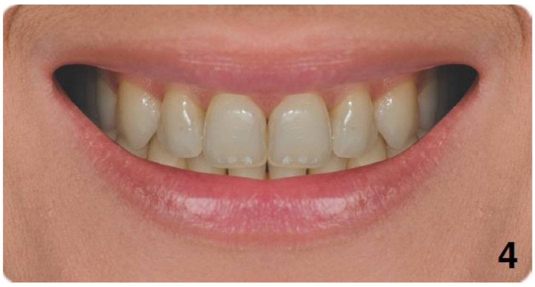

E: +4 mm

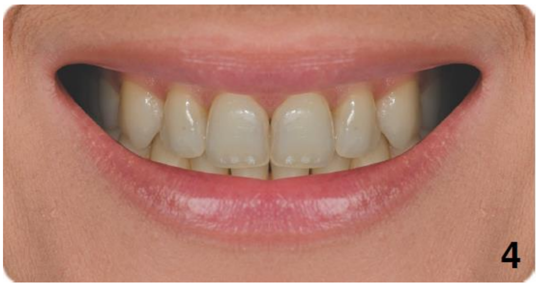

Smile 5. The vertical position of the cusps of the canines is shifted coronally

A: unmodified

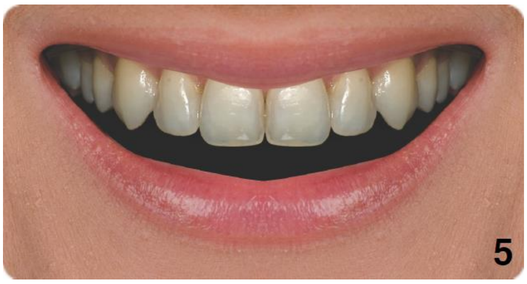

B: -1mm

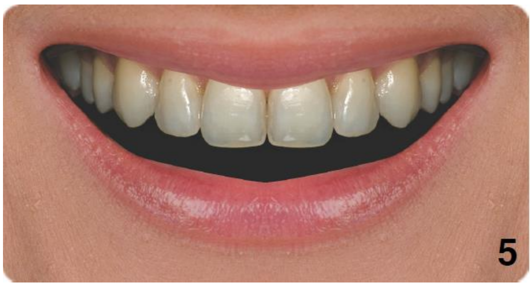

C: -2mm

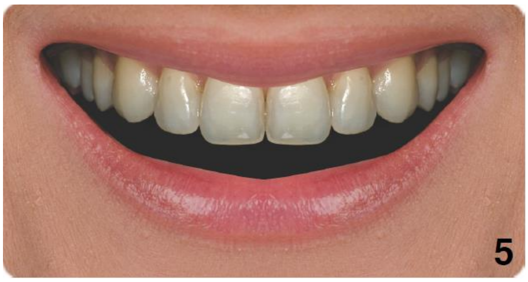

D: cusps at arch level

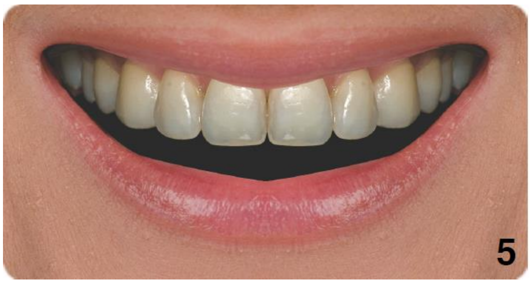

E: arch level -1 mm

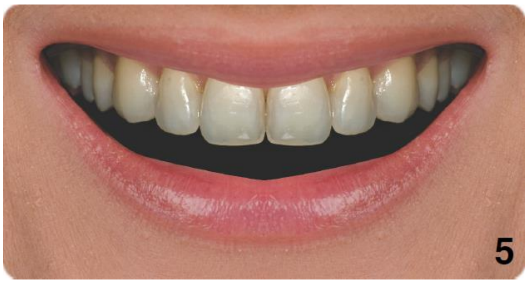

Smile 6. The zenith of the front teeth changes

A: unmodified

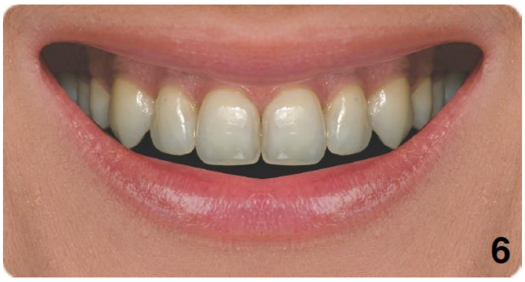

B: arching upward from the midline

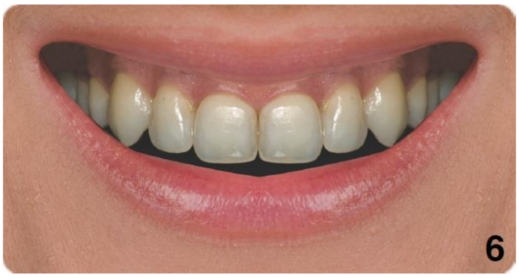

C: arching downward from the midline

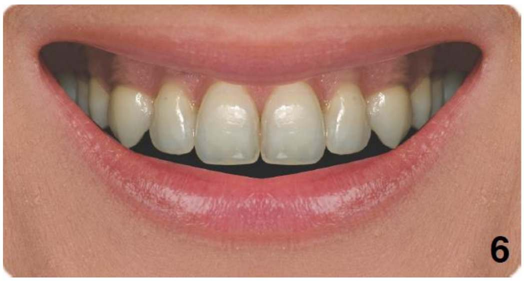

D: first upward then downward

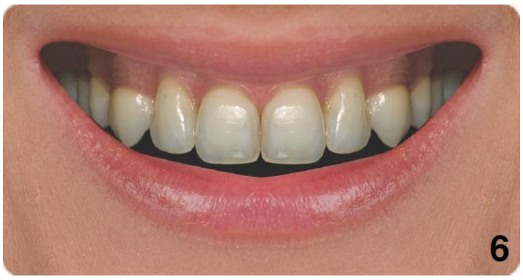

E: horizontal

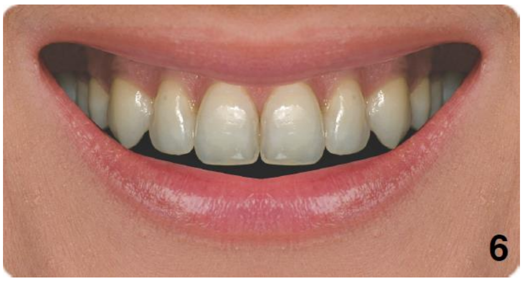

Smile 7. The length of frontal interdental papillae changes

A: unmodified

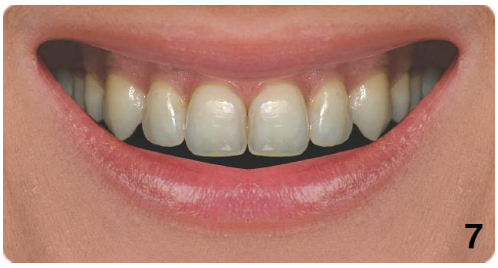

B: - 1 mm between the central incisors, + 1 mm between the central and lateral incisors, + 1 mm between the lateral incisors and canines

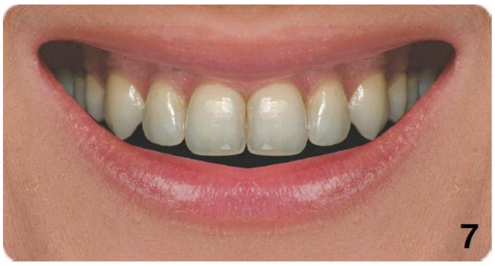

C: - 1 mm in all positions

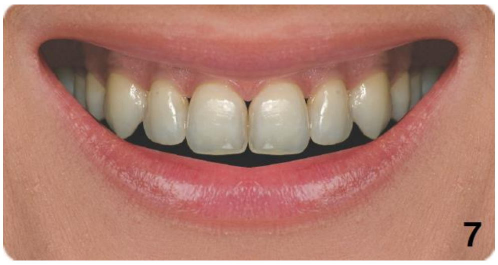

D: + 1 mm between the central incisors, - 1 mm between the central and lateral incisors, + 1 mm between the lateral incisors and canines

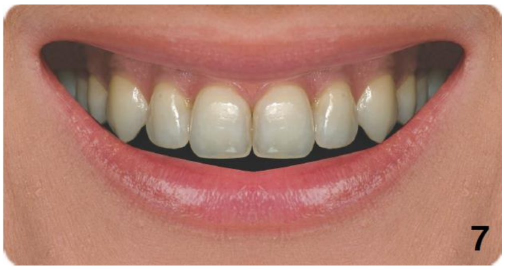

E: - 1 mm between the central incisors, + 1 mm between the central and lateral incisors, - 1 mm between the lateral incisors and canines

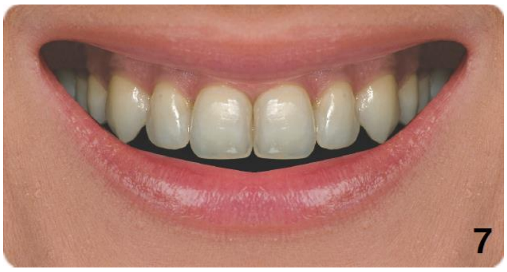

Smile 8. Midline shift

A: 2 mm to left

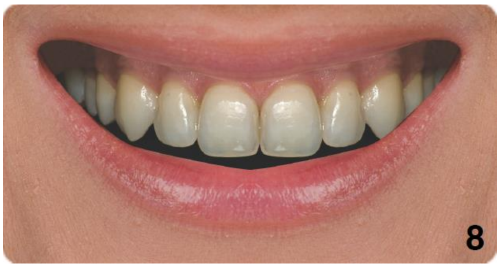

B: 1 mm to left

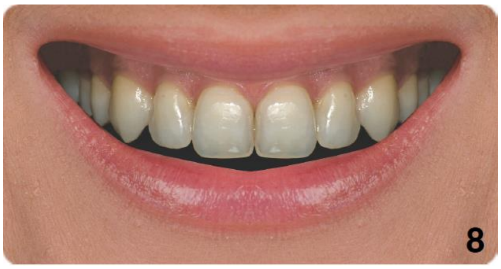

C: unmodified

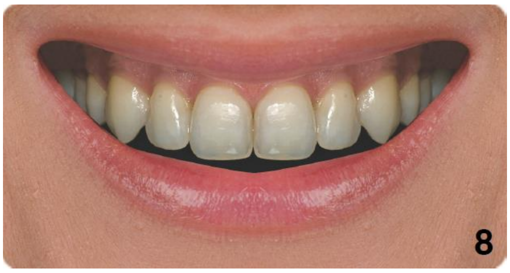

D: 1 mm to right

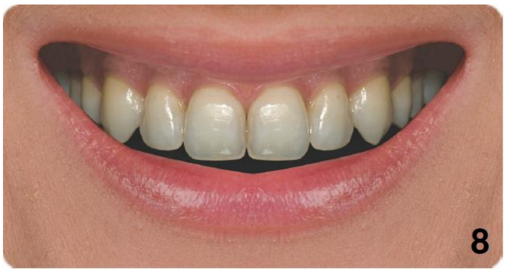

E: 2 mm to right

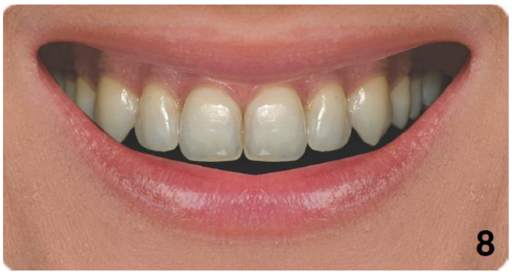

Smile 9. The position of the commissures changes

A: unmodified

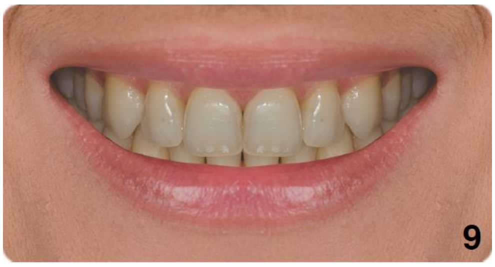

C: 1 mm downward

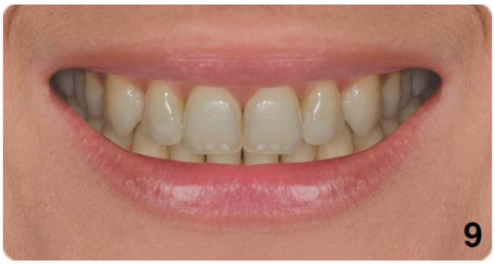

D: 2 mm downward

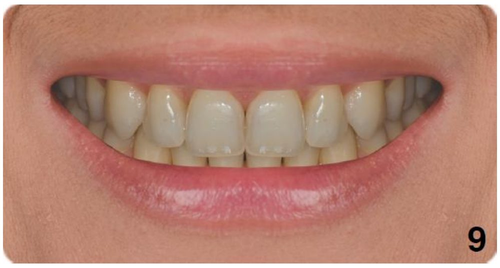

E: 3 mm downward

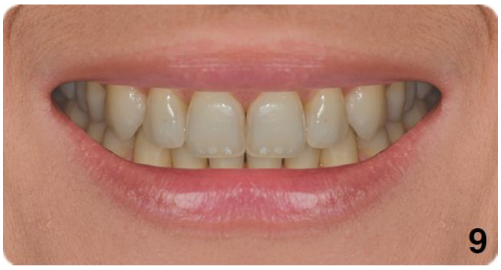

B: 4 mm downward

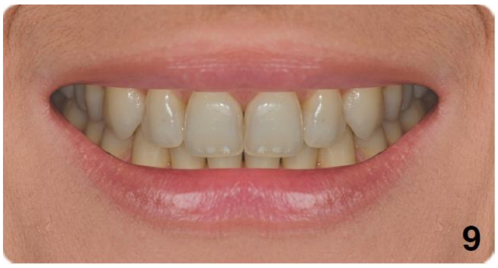

Smile 10. The position of the arches changes (as related to the lips)

A: 1 mm upward

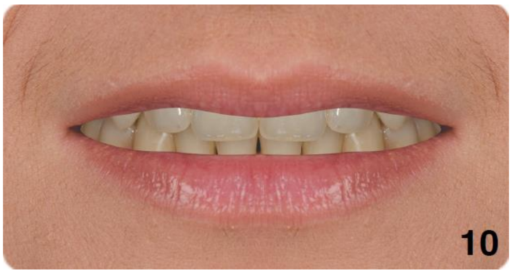

B: 0.5 mm upward

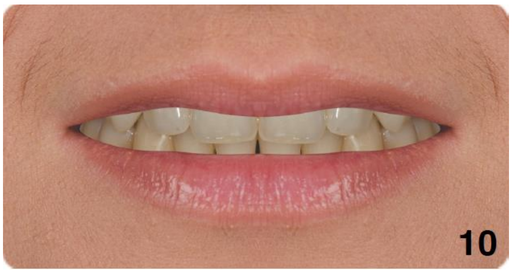

C: unmodified

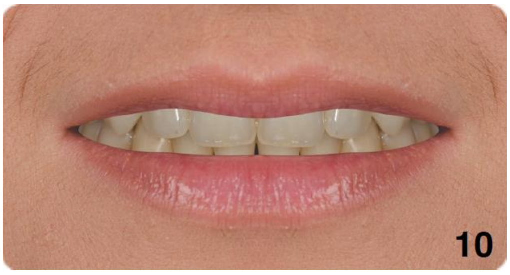

D: 0.5 mm downward

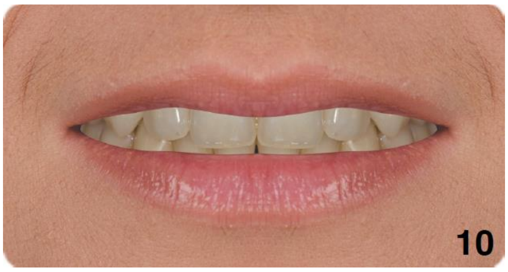

E: 1 mm downward

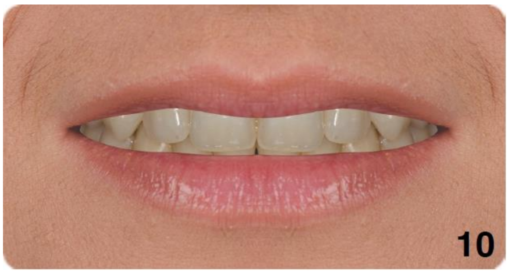

Supplement: S2 Data — (PDF) [file pone.0230182.s002.pdf]
